# Commentary: Long Non-Coding RNA Gene Polymorphisms and Their Expression Levels in Patients With Rheumatoid Arthritis

**DOI:** 10.3389/fimmu.2021.801266

**Published:** 2021-12-09

**Authors:** Jorge Hernández-Bello, Christian Johana Baños-Hernández, José Francisco Muñoz-Valle

**Affiliations:** Institute for Research in Biomedical Sciences (IICB), University Center for Health Sciences, University of Guadalajara, Guadalajara, Mexico

**Keywords:** ANRIL, lnc-DC, single-nucleotide polymorphism, rheumatoid arthritis, ZFAS1, MALAT1

## Introduction

We read the paper by Zhang et al. (1) with interest. The authors report on a study that evaluated the association of four long noncoding RNA (lncRNA) (ANRIL, lnc-DC, MALAT1, ZFAS1) gene single-nucleotide polymorphisms (SNPs) with susceptibility to rheumatoid arthritis (RA) patients, as well as their expression levels. They concluded that *ANRIL*, *lnc-DC*, *MALAT1*, and *ZFAS1* gene SNPs were not associated with RA susceptibility after false discovery rate (FDR) correction, while altered ANRIL, lnc-DC, MALAT1, and ZFAS1 levels in RA patients suggested that these lncRNAs might play a role in RA.

After carefully reading, we identified some mistakes in the odds ratio (OR) calculations in **Table 1** “Genotypes and alleles frequencies of lncRNAs genes polymorphisms in RA patients and normal controls” [sic]. Likewise, there are inconsistencies in the genetic models.

**Table 1:** Correction: Genotypes and allele frequencies of lncRNA gene polymorphisms in RA patients and normal controls.

SNP	Analyze model		RA (N = 660) n (%)	Control (N = 710) n (%)	p value	OR (95% CI)
***ANRIL***						
rs1412830	Genotypes	TT	13 (1.97)	3 (0.42)	**0.008**	**4.66 (1.32–16.45)**
		CT	119 (18.03)	139 (19.58)	0.55	0.92 (0.70–1.21)
		CC	528 (80.00)	568 (80.00)	Reference	
	Alleles	T	145 (10.98)	145 (10.21)	0.51	1.08 (0.85–1.38)
		C	1,175 (89.02)	1,275 (89.79)	Reference	
	Dominant model	TT+CT	132 (20.00)	142 (20.00)	1	1.00 (0.76–1.30)
		CC	528 (80.00)	568 (80.00)	Reference	
	Recessive model	TT	13 (1.97)	3 (0.42)	**0.007**	**4.73 (1.29–16.69)**
		CC+CT	647 (98.03)	707 (99.58)	Reference	
rs944796	Genotypes	GG	11 (1.67)	31 (4.37)	**0.006**	**0.38 (0.19–0.78)**
		GC	238 (36.06)	230 (32.39)	0.28	1.13 (0.90–1.41)
		CC	411 (62.27)	449 (63.24)	Reference	
	Alleles	G	260 (19.70)	292 (20.56)	0.57	0.95 (0.78–1.14)
		C	1,060 (80.30)	1,128 (79.44)	Reference	
	Dominant model	GG+GC	249 (37.73)	261 (36.76)	0.71	1.04 (0.83–1.30)
		CC	411 (62.27)	449 (63.24)	Reference	
	Recessive model	GG	11 (1.67)	31 (4.37)	**0.003**	**0.37 (0.18–0.74)**
		CC+GC	649 (98.33)	679 (95.63)	Reference	
rs61271866	Genotypes	AA	25 (3.79)	26 (3.66)	0.98	1.00 (0.57–1.76)
		TA	185 (28.03)	214 (30.14	0.39	0.9 (0.71–1.14)
		TT	450 (68.18)	470 (66.20)	Reference	
	Alleles	A	235 (17.80)	266 (18.73)	0.52	0.94 (0.77–1.14)
		T	1,085 (82.20)	1,154 (81.27)	Reference	
	Dominant model	AA+TA	210 (31.82)	240 (33.80)	0.43	0.91 (0.72–1.14)
		TT	450 (68.18)	470 (66.20)	Reference	
	Recessive model	AA	25 (3.79)	26 (3.66)	0.90	1.03 (0.59–1.81)
		TT+TA	635 (96.21)	684 (96.34)	Reference	
rs2518723	Genotypes	TT	111 (16.82)	133 (18.73)	0.26	0.83 (0.61–1.14)
		CT	326 (49.39)	353 (49.72)	0.53	0.92 (0.73–1.17)
		CC	223 (33.79)	224 (31.55)	Reference	
	Alleles	T	548 (41.52)	619 (43.59)	0.27	0.91 (0.78–1.06)
		C	772 (58.48)	801 (56.41)	Reference	
	Dominant model	TT+CT	437 (66.21)	486 (68.45)	0.37	0.90 (0.72–1.13)
		CC	223 (33.79)	224 (31.55)	Reference	
	Recessive model	TT	111 (16.82)	133 (18.73)	0.35	0.87 (0.66–1.15)
		CC+CT	549 (83.18)	577 (81.27)	Reference	
rs3217992	Genotypes	TT	160 (24.24)	152 (21.41)	0.11	1.27 (0.93–1.72)
		CT	338 (51.21)	362 (50.99)	0.34	1.13 (0.87–1.45)
		CC	162 (24.55)	196 (27.61)	Reference	
	Alleles	T	658 (49.85)	666 (46.90)	0.12	1.12 (0.96–1.30)
		C	662 (50.15)	754 (53.10)	Reference	
	Dominant model	TT+CT	498 (75.45)	514 (72.39)	0.19	1.17 (0.92–1.49)
		CC	162 (24.55)	196 (27.61)	Reference	
	Recessive model	TT	160 (24.24)	152 (21.41)	0.21	1.17 (0.91–1.51)
		CC+CT	500 (75.76)	558 (78.59)	Reference	
***Lnc-DC***						
rs7217280	Genotypes	AA	3 (0.45)	4 (0.56)	0.74	0.78 (0.17–3.50)
		GA	52 (7.88)	77 (10.85)	0.059	0.70 (0.48–1.01)
		GG	605 (91.67)	629 (88.59)	Reference	
	Alleles	A	58 (4.39)	85 (5.99)	0.06	0.72 (0.51–1.01)
		G	1,262 (95.61)	1,335 (94.01)	Reference	
	Dominant model	AA+GA	55 (8.33)	81 (11.41)	0.057	0.70 (0.49–1.01)
		GG	605 (91.67)	629 (88.59)	Reference	
	Recessive model	AA	3 (0.45)	4 (0.56)	0.77	0.80 (0.18–3.61)
		GG+GA	657 (99.55)	706 (99.44)	Reference	
rs10515177	Genotypes	GG	4 (0.61)	5 (0.70)	0.79	0.83 (0.22–3.13)
		AG	94 (14.24)	117 (16.48)	0.24	0.84 (0.62–1.12)
		AA	562 (85.15)	588 (82.82)	Reference	
	Alleles	G	102 (7.73)	127 (8.94)	0.25	0.85 (0.65–1.11)
		A	1,218 (92.27)	1,293 (91.06)	Reference	
	Dominant model	GG+AG	98 (14.85)	122 (17.18)	0.23	0.84 (0.62–1.12)
		AA	562 (85.15)	588 (82.82)	Reference	
	Recessive model	GG	4 (0.61)	5 (0.70)	0.82	0.86 (0.23–3.21)
		AA+AG	656 (99.39)	705 (99.30)	Reference	
***MALAT1***						
rs619586	Genotypes	GG	6 (0.91)	4 (0.56)	0.44	1.63 (0.46–5.83)
		AG	111 (16.82)	113 (15.92)	0.63	1.07 (0.80–1.42)
		AA	543 (82.27)	593 (83.52)	Reference	
	Alleles	G	123 (9.32)	121 (8.52)	0.46	1.10 (0.84–1.43)
		A	1,197 (90.68	1,299 (91.48)	Reference	
	Dominant model	GG+AG	117 (17.73)	117 (16.48)	0.53	1.09 (0.82–1.44)
		AA	543 (82.27)	593 (83.25)	Reference	
	Recessive model	GG	6 (0.91)	4 (0.56)	0.45	1.61 (0.45–5.76)
		AA+AG	654 (99.09)	706 (99.44)	Reference	
rs4102217	Genotypes	CC	20 (3.03)	13 (1.83)	0.21	1.55 (0.76–3.16)
		GC	154 (23.33)	205 (28.87)	**0.02**	**0.76 (0.59–0.97)**
		GG	486 (73.64	492 (69.30)	Reference	
	Alleles	C	194 (14.70)	231 (16.27)	0.25	0.88 (0.72–1.09)
		G	1,126 (85.30)	1,189 (83.73)	Reference	
	Dominant model	CC+GC	174 (26.36)	218 (30.70)	0.07	0.80 (0.63–1.02)
		GG	486 (73.64)	492 (69.30)	Reference	
	Recessive model	CC	20 (3.03)	13 (1.83)	0.14	1.67 (0.82–3.39)
		GG+GC	640 (96.97)	697 (98.17)	Reference	
rs591291	Genotypes	TT	124 (18.79)	132 (18.59)	0.55	0.91 (0.67–1.23)
		CT	298 (45.15)	347 (48.87)	0.13	0.83 (0.65–1.05)
		CC	238 (36.06)	231 (32.53)	Reference	
	Alleles	T	546 (41.36)	611 (43.03)	0.37	0.93 (0.80–1.08)
		C	774 (58.64)	809 (56.97)	Reference	
	Dominant model	TT+CT	422 (63.94	479 (67.46)	0.16	0.85 (0.68–1.06)
		CC	238 (36.06)	231 (32.53)	Reference	
	Recessive model	TT	124 (18.79)	132 (18.59)	0.92	1.01 (0.77–1.32)
		CC+CT	536 (81.21)	578 (81.41)	Reference	
rs11227209	Genotypes	GG	3 (0.45)	3 (0.42)	0.93	1.07 (0.21–5.33)
		CG	71 (10.76)	79 (11.13)	0.82	0.96 (0.68–1.35)
		CC	586 (88.79)	628 (88.45)	Reference	
	Alleles	G	77 (5.83)	85 (5.99)	0.86	0.97 (0.70–1.33)
		C	1,243 (94.17)	1,335 (94.01)	Reference	
	Dominant model	GG+CG	74 (11.21)	82 (11.55)	0.84	0.96 (0.69–1.35)
		CC	586 (88.79)	628 (88.45)	Reference	
	Recessive model	GG	3 (0.45)	3 (0.42)	0.92	1.07 (0.21–5.35)
		CC+CG	657 (99.55)	707 (99.58)	Reference	
rs35138901	Genotypes	CC	4 (0.61)	2 (0.28)	0.37	2.10 (0.38–11.54)
		TC	93 (14.09)	115 (16.20)	0.28	0.85 (0.63–1.14)
		TT	563 (85.30)	593 (83.52)	Reference	
	Alleles	C	101 (7.65)	119 (8.38)	0.48	0.90 (0.68–1.19)
		T	1,219 (92.35)	1,301 (91.62)	Reference	
	Dominant model	CC+TC	97 (14.70)	117 (16.48	0.36	0.87 (0.65–1.17)
		TT	563 (85.30)	593 (83.52)	Reference	
	Recessive model	CC	4 (0.61)	2 (0.28)	0.36	2.15 (0.39–11.82)
		TT+TC	656 (99.39)	708 (99.72)	Reference	
***ZFAS1***						
rs237742	Genotypes	TT	91 (13.79)	104 (14.65)	0.93	1.01 (0.72–1.40)
		CT	322 (48.79)	320 (45.07)	0.19	1.16 (0.92–1.46)
		CC	247 (37.42)	286 (40.28)	Reference	
	Alleles	T	504 (38.18)	528 (37.18)	0.58	1.04 (0.89–1.21)
		C	816 (61.82)	892 (62.82)	Reference	
	Dominant model	TT+CT	413 (62.58)	424 (59.72)	0.27	1.12 (0.90–1.40)
		CC	247 (37.42)	286 (40.28)	Reference	
	Recessive model	TT	91 (13.79)	104 (14.65)	0.64	0.93 (0.68–1.26)
		CC+CT	569 (86.21)	606 (85.35)	Reference	
rs73116127	Genotypes	AA	1 (0.15)	3 (0.42)	0.33	0.34 (0.03–3.35)
		GA	109 (16.52)	133 (18.73)	0.27	0.85 (0.64–1.13)
		GG	550 (83.33)	574 (80.85)	Reference	
	Alleles	A	111 (8.41)	139 (9.79)	0.21	0.84 (0.65–1.09)
		G	1,209 (91.59)	1,281 (90.21)	Reference	
	Dominant model	AA+GA	110 (16.67)	136 (19.15)	0.23	0.84 (0.64–1.11)
		GG	550 (83.33)	574 (80.85)	Reference	
	Recessive model	AA	1 (0.15)	3 (0.42)	0.35	0.35 (0.03–3.44)
		GG+GA	659 (99.85)	707 (99.58)	Reference	
rs6125607	Genotypes	TT	74 (11.21)	48 (6.76)	**0.004**	**1.75 (1.18–2.60)**
		CT	277 (41.97)	310 (43.66)	0.87	1.01 (0.81–1.27)
		CC	309 (46.82)	352 (49.58)	Reference	
	Alleles	T	425 (32.20)	406 (28.59)	**0.04**	**1.18 (1.01–1.39)**
		C	895 (67.80)	1,014 (71.41)	Reference	
	Dominant model	TT+CT	351 (53.18)	358 (50.42)	0.30	1.11 (0.90–1.38)
		CC	309 (46.82)	352 (49.58)	Reference	
	Recessive model	TT	74 (11.21)	48 (6.76)	**0.003**	**1.74 (1.19–2.54)**
		CC+CT	586 (88.78)	662 (93.23)	Reference	
rs6125608	Genotypes	GG	9 (1.36)	11 (1.55)	0.70	0.84 (0.34–2.04)
		AG	125 (18.94)	158 (22.25)	0.12	0.81 (0.62–1.05)
		AA	526 (79.70)	541 (76.20)	Reference	
	Alleles	G	143 (10.83)	180 (12.68	0.13	0.83 (0.66–1.05)
		A	1,177 (89.17)	1,240 (87.32)	Reference	
	Dominant model	GG+AG	134 (20.30)	169 (23.80)	0.11	0.81 (0.63–1.05)
		AA	526 (79.70)	541 (76.20)	Reference	
	Recessive model	GG	9 (1.36)	11 (1.55)	0.77	0.87 (0.36–2.13)
		AA+AG	651 (98.64)	699 (98.45)	Reference	

Bold values denote statistical significance at the p < 0.05 level.

CI, confidence interval; lncRNA, long noncoding RNA; OR, odds ratio; RA, rheumatoid arthritis; SNP, single-nucleotide polymorphism.

ANRIL, antisense non-coding RNA in the INK4 locus); Lnc-DC, Lnc-RNA in dendritic cell; MALAT1, metastasis-associated lung adenocarcinoma transcript-1; zinc finger antisense 1.

In general, we find that the OR values were calculated and interpreted in an inappropriate way. This is very noticeable when analyzing the frequency of genotypes in cases and controls. For example, for rs1412830, the authors reported an OR = 0.214 (0.060–0.761), p = 0.017, for the TT genotype. That OR value suggests that this genotype is a protective or lower risk factor. However, the TT genotype has a higher prevalence in patients than that in controls (1.97% vs. 0.42%, respectively); therefore, the OR value should be >1.

Regarding genetic models, the authors maintain the same mistake in the OR values. Taking rs1412830 as an example, we once again observed that there is a higher frequency of the TT genotype in patients than that in controls (1.97% vs. 0.42%, respectively). However, the authors reported an OR value = 0.211 (0.059–0.750), p = 0.016, but they describe it as a risk factor. We correctly calculate the OR values for your consideration (**Table 1**).

We recommend that the authors (1) recalculate these data appropriately (2) in order to be able to rediscuss all their results. Also, we suggest that they corroborate the OR values in Table 3 (1), which we could not analyze due to lack of data.

## Author Contributions

JH-B wrote this commentary and analyzed the data. CB-H and JM-V performed the analysis. All authors contributed to the article and approved the submitted version.

## Conflict of Interest

The authors declare that the research was conducted in the absence of any commercial or financial relationships that could be construed as a potential conflict of interest.

## Publisher’s Note

All claims expressed in this article are solely those of the authors and do not necessarily represent those of their affiliated organizations, or those of the publisher, the editors and the reviewers. Any product that may be evaluated in this article, or claim that may be made by its manufacturer, is not guaranteed or endorsed by the publisher.
